# Supplementary figures and images for: Downregulation of 5-hydroxymethylcytosine is associated with the progression of cervical intraepithelial neoplasia
Source: PLoS One. 2020 Nov 3;15(11):e0241482. doi: 10.1371/journal.pone.0241482 (PMC7608920; doi:10.1371/journal.pone.0241482)

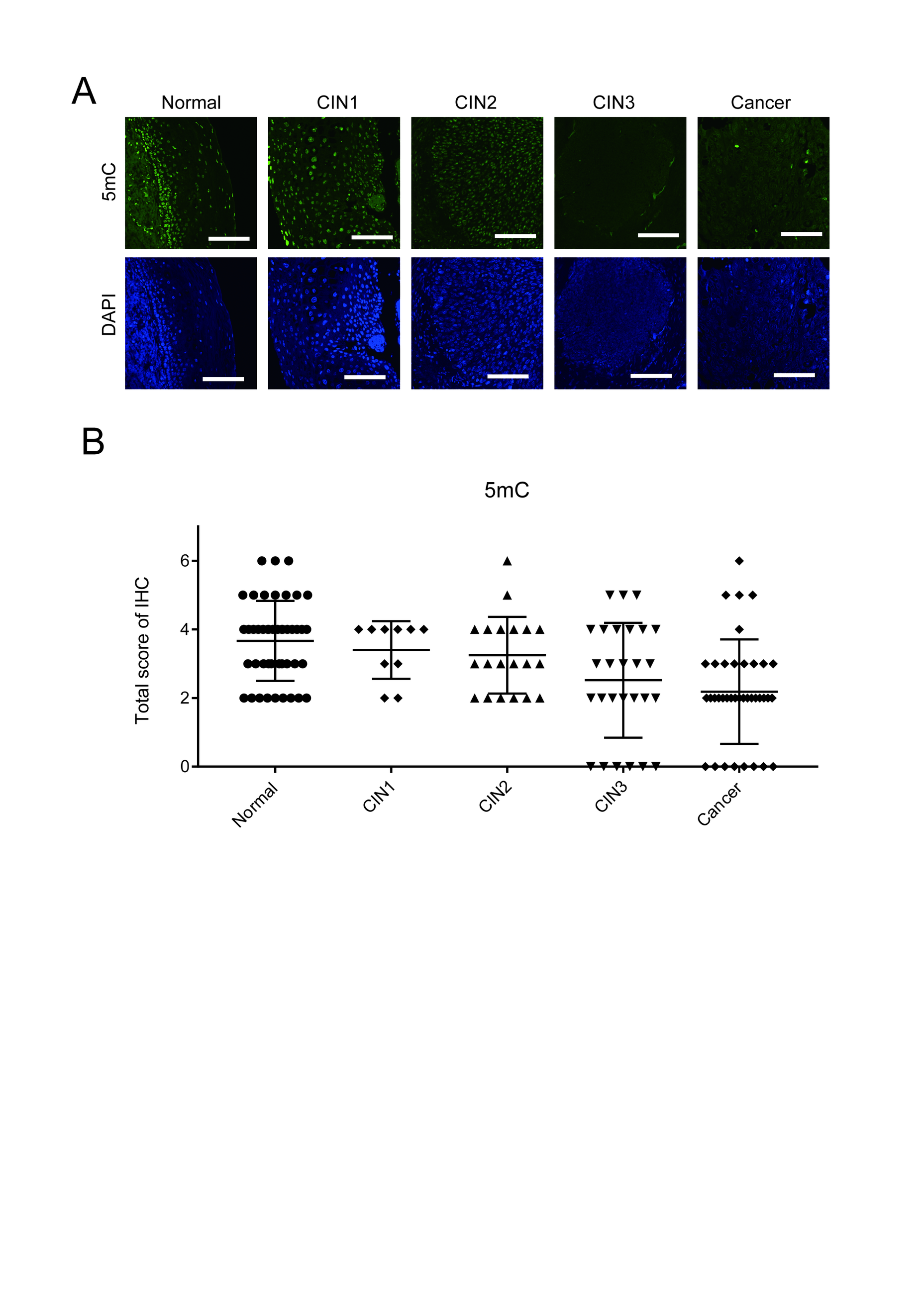

Supplement: S1 Fig — (A) Representative images of IHC staining of 5mC with DAPI. Scale bar, 100 μm. (B) Dot plot shows the IHC scores for the level of 5hmC in normal cervical epithelium, CIN1, CIN2, CIN3 and cancer. Error bars indicate the standard deviation. (TIF) [file pone.0241482.s001.tif]

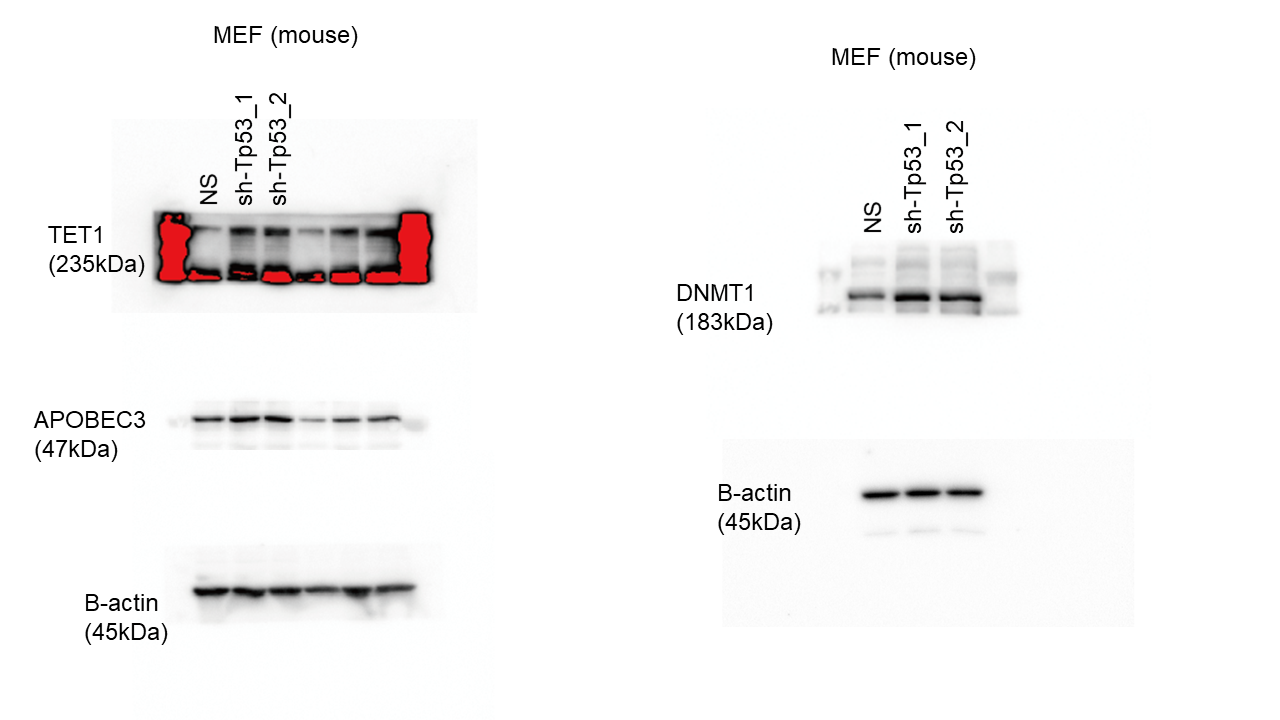

Supplement: S1 Raw data — (TIF) [file pone.0241482.s007.tif]

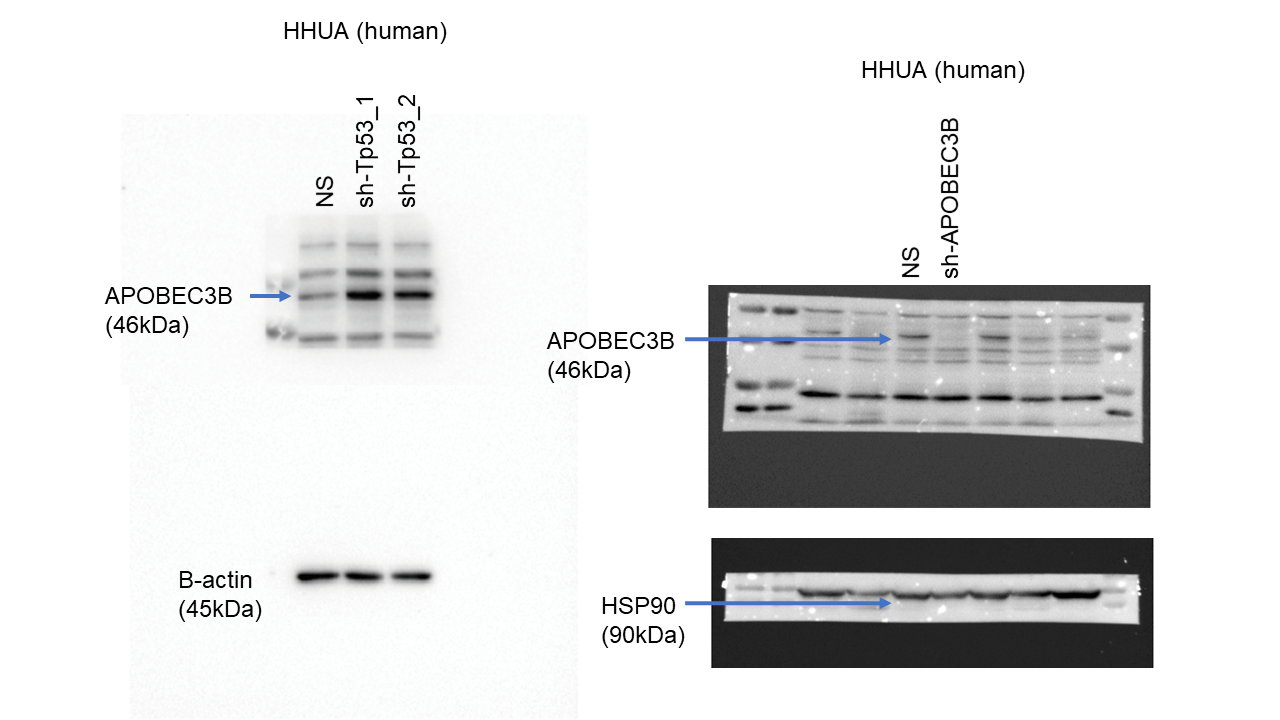

Supplement: S2 Raw data — (TIF) [file pone.0241482.s008.tif]

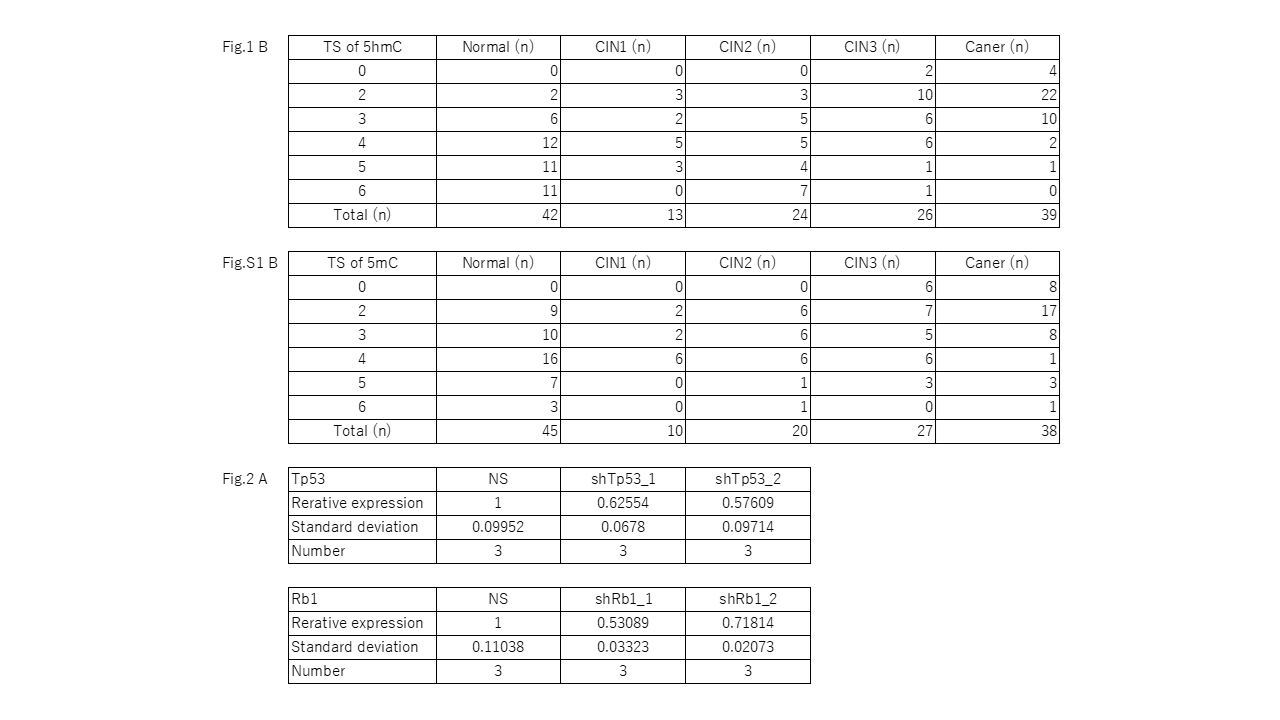

Supplement: S3 Raw data — (TIF) [file pone.0241482.s009.tif]

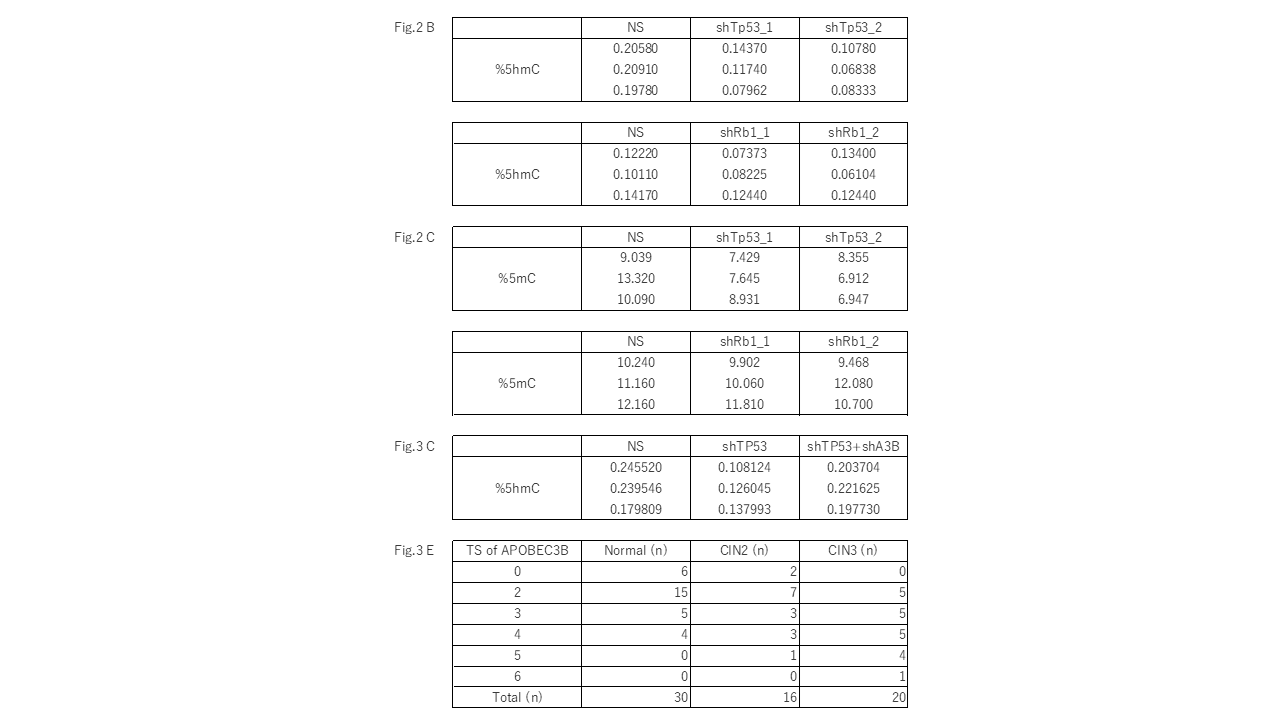

Supplement: S4 Raw data — (TIF) [file pone.0241482.s010.tif]
