# Supplementary material for: Downregulation of 5-hydroxymethylcytosine is associated with the progression of cervical intraepithelial neoplasia
Source: PLoS One. 2020 Nov 3;15(11):e0241482. doi: 10.1371/journal.pone.0241482 (PMC7608920; doi:10.1371/journal.pone.0241482)
Supplement: S1 Table — (DOCX) [file pone.0241482.s002.docx]

| **S1 Table. Antibodies used for immunohistochemistry, ELISA and western blot analysis.** | | | |
| --- | --- | --- | --- |
| Antigen | Source | Catalog No. | Dilution, Application |
| 5hmC | Active Motif | 39999 | 1/500, IHC |
| 5hmC | Zymo research | A4001-25 | 1/1000, ELISA |
| 5mC | Active Motif | 39649 | 1/500, IHC |
| 5mC | Zymo research | A3001-30 | 1/1000, ELISA |
| DNMT1 | Novus Biologicals | NB100-56519 | 1/2000, WB |
| TET1 | Abcam | ab191698 | 1/500, WB |
| APOBEC3B | Gene Tex | GTX17214 | 1/250, WB |
| HSP90 | BD Transduction Laboratories | 610419 | 1/1000, WB |
| β-actin | Santa Cruz Biotechnology | sc-81178 | 1/1000, WB |
