# Supplementary material for: Downregulation of 5-hydroxymethylcytosine is associated with the progression of cervical intraepithelial neoplasia
Source: PLoS One. 2020 Nov 3;15(11):e0241482. doi: 10.1371/journal.pone.0241482 (PMC7608920; doi:10.1371/journal.pone.0241482)
Supplement: S2 Table — (DOCX) [file pone.0241482.s003.docx]

| **S2 Table. Scoring of system for IHC immunohistochemical staining.** | | | | |
| --- | --- | --- | --- | --- |
| Score for proportion of  positive cells | | Score for staining  intensity | | Total score |
| 0 | 0% | 0 | negative |  |
| 1 | 1–33% | 1 | low | Sum of the two scores |
| 2 | 34–66% | 2 | moderate |  |
| 3 | 67–100% | 3 | high |  |
