# Supplementary material for: Downregulation of 5-hydroxymethylcytosine is associated with the progression of cervical intraepithelial neoplasia
Source: PLoS One. 2020 Nov 3;15(11):e0241482. doi: 10.1371/journal.pone.0241482 (PMC7608920; doi:10.1371/journal.pone.0241482)
Supplement: S3 Table — (DOCX) [file pone.0241482.s004.docx]

| **S3 Table. Target sequences of the shRNAs.** | | |
| --- | --- | --- |
| Species | shRNA | Target sequence |
| mouse | shTp53_1 | TATTGTTTCCAAAGAGAAGCG |
|  | shTp53_2 | ATTCTGTTTCTAGAAATGACG |
|  | shRb1_1 | CCGTGGATTCTGAACGTACTT |
|  | shRb1_2 | CCACTCGAACACGAATGCAAA |
|  |  |  |
| human | shTP53_1 | CGGCGCACAGAGGAAGAGAAT |
|  | shTP53_2 | CACCATCCACTACAACTACAT |
|  | shAPOBEC3B_1 | GACCTACTTGTGCTATGAGGT |
|  | shAPOBEC3B_2 | CCAGGTGTATTTCAAGCCTCA |
