# Supplementary material for: Downregulation of 5-hydroxymethylcytosine is associated with the progression of cervical intraepithelial neoplasia
Source: PLoS One. 2020 Nov 3;15(11):e0241482. doi: 10.1371/journal.pone.0241482 (PMC7608920; doi:10.1371/journal.pone.0241482)
Supplement: S4 Table — (DOCX) [file pone.0241482.s005.docx]

| **S4 Table. Primer sequences used in qRT-PCR experiments.** | | |  |
| --- | --- | --- | --- |
| Species | Gene symbol | Sequences | Base pairs |
| Mouse | *Tp53* | F: GCAACTATGGCTTCCACCTG | 82 |
|  |  | R: TTATTGAGGGGAGGAGAGTACG |  |
|  | *Rb1* | F: CATCTAATGGACTTCCAGAG | 218 |
|  |  | R: CATAACAGTCCTAACTGGAG |  |
|  | *Oaz1* | F: GCACTGCTGAGAGCAAGATG | 213 |
|  |  | R: ATAGGCAGTGCCAAGGAAGA |  |
|  | *Rps29* | F: GGATCCTCAATAGCCACTGC | 150 |
|  |  | R: TACAGCAGTGGAGGGAGACC |  |
|  |  |  |  |
| Human | *TP53* | F: CCTCAGCATCTTATCCGAGTGG | 128 |
|  |  | R: TGGATGGTGGTACAGTCAGAGC |  |
