# Supplementary material for: Downregulation of 5-hydroxymethylcytosine is associated with the progression of cervical intraepithelial neoplasia
Source: PLoS One. 2020 Nov 3;15(11):e0241482. doi: 10.1371/journal.pone.0241482 (PMC7608920; doi:10.1371/journal.pone.0241482)
Supplement: S5 Table — (DOCX) [file pone.0241482.s006.docx]

| **S5 Table. Statistical analysis of 5mC levels in normal cervical epithelia and cervical tumors** | | | | |
| --- | --- | --- | --- | --- |
| Tukey's multiple comparisons test | Mean Diff. | 95.00% CI of diff. | Summary | Adjusted P Value |
| Normal vs. CIN1 | 0.2667 | -1.045 to 1.578 | ns | 0.9802 |
| Normal vs. CIN2 | 0.4167 | -0.5917 to 1.425 | ns | 0.7836 |
| Normal vs. CIN3 | 1.148 | 0.2348 to 2.062 | ** | 0.0061 |
| Normal vs. Cancer | 1.482 | 0.6558 to 2.309 | **** | <0.0001 |
| CIN1 vs. CIN2 | 0.15 | -1.303 to 1.603 | ns | 0.9985 |
| CIN1 vs. CIN3 | 0.8815 | -0.5075 to 2.27 | ns | 0.4044 |
| CIN1 vs. Cancer | 1.216 | -0.1178 to 2.549 | ns | 0.092 |
| CIN2 vs. CIN3 | 0.7315 | -0.3755 to 1.838 | ns | 0.3624 |
| CIN2 vs. Cancer | 1.066 | 0.02925 to 2.102 | * | 0.0406 |
| CIN3 vs. Cancer | 0.3343 | -0.6101 to 1.279 | ns | 0.8644 |
